# Regulation of mRNA Stability During Bacterial Stress Responses

**DOI:** 10.3389/fmicb.2020.02111

**Published:** 2020-09-09

**Authors:** Diego A. Vargas-Blanco, Scarlet S. Shell

**Affiliations:** ^1^Department of Biology and Biotechnology, Worcester Polytechnic Institute, Worcester, MA, United States; ^2^Program in Bioinformatics and Computational Biology, Worcester Polytechnic Institute, Worcester, MA, United States

**Keywords:** ribonucleic acid, stress response, carbon starvation, nutrient starvation, hypoxia, mRNA degradation, mRNA stability, bacteria

## Abstract

Bacteria have a remarkable ability to sense environmental changes, swiftly regulating their transcriptional and posttranscriptional machinery as a response. Under conditions that cause growth to slow or stop, bacteria typically stabilize their transcriptomes in what has been shown to be a conserved stress response. In recent years, diverse studies have elucidated many of the mechanisms underlying mRNA degradation, yet an understanding of the regulation of mRNA degradation under stress conditions remains elusive. In this review we discuss the diverse mechanisms that have been shown to affect mRNA stability in bacteria. While many of these mechanisms are transcript-specific, they provide insight into possible mechanisms of global mRNA stabilization. To that end, we have compiled information on how mRNA fate is affected by RNA secondary structures; interaction with ribosomes, RNA binding proteins, and small RNAs; RNA base modifications; the chemical nature of 5′ ends; activity and concentration of RNases and other degradation proteins; mRNA and RNase localization; and the stringent response. We also provide an analysis of reported relationships between mRNA abundance and mRNA stability, and discuss the importance of stress-associated mRNA stabilization as a potential target for therapeutic development.

## Introduction

Bacterial adaptation to stress is orchestrated by complex responses to specific environmental stimuli, capable of rapidly regulating transcription, transcript degradation, and translation, which increases the organism’s survival opportunities. Historically, regulation mechanisms for transcriptional and translational pathways have been the most studied, providing insight into the genes and protein products needed for bacterial adaptation to unfavorable growth environments. These findings have been key for our understanding of bacterial biology, allowing us, for example, to develop tools to tune bacterial machinery for biotechnology processes (such as Tao et al., 2011; Courbet et al., 2015; Daeffler et al., 2017; Martinez et al., 2017; Riglar et al., 2017), and to discover and develop new antibacterial drugs (for example, Yarmolinsky and Haba, 1959; Wolfe and Hahn, 1965; Maggi et al., 1966; Olson et al., 2011). However, the role of RNA degradation in stress responses is not well understood.

**FIGURE 1:**
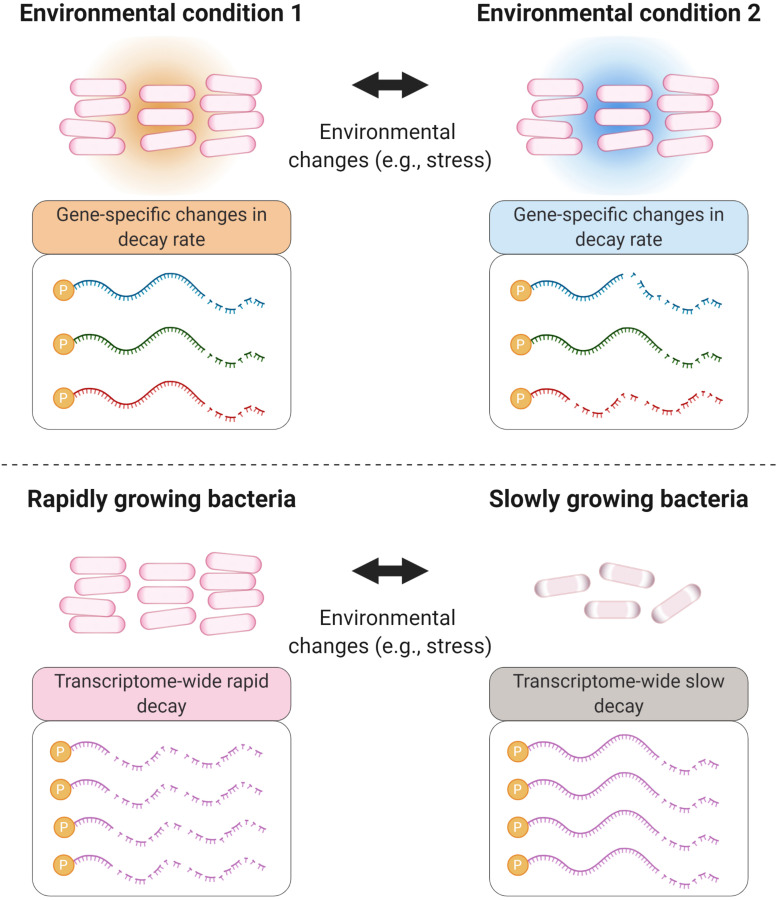
Environmental changes cause mRNA degradation rates to change in both global and gene-specific ways. Bacterial adaptation to many stressors, and other changes in environment, involve modulation of degradation rates of specific transcripts encoding proteins relevant to the changing conditions (top panel). Some stressors, particularly those causing severe energy stress, trigger global stabilization of the mRNA pool (bottom panel). These scenarios are not mutually exclusive; stressors that cause global transcriptome stabilization typically also cause gene-specific changes in relative degradation rates.

Modulation of mRNA degradation has been associated with various stress conditions in bacteria, such as temperature changes, growth rate, nutrient starvation, and oxygen limitation (see Table 1). Transcript stability – also referred as mRNA or transcript half-life – was shown to be globally altered in response to some stressors, while in other cases, gene-specific modulation of transcript stability contributes to specific expression changes that bacteria need to adapt to and survive in new environments (Figure 1).

**TABLE 1:** Transcriptome-wide studies on mRNA half-life in bacteria.

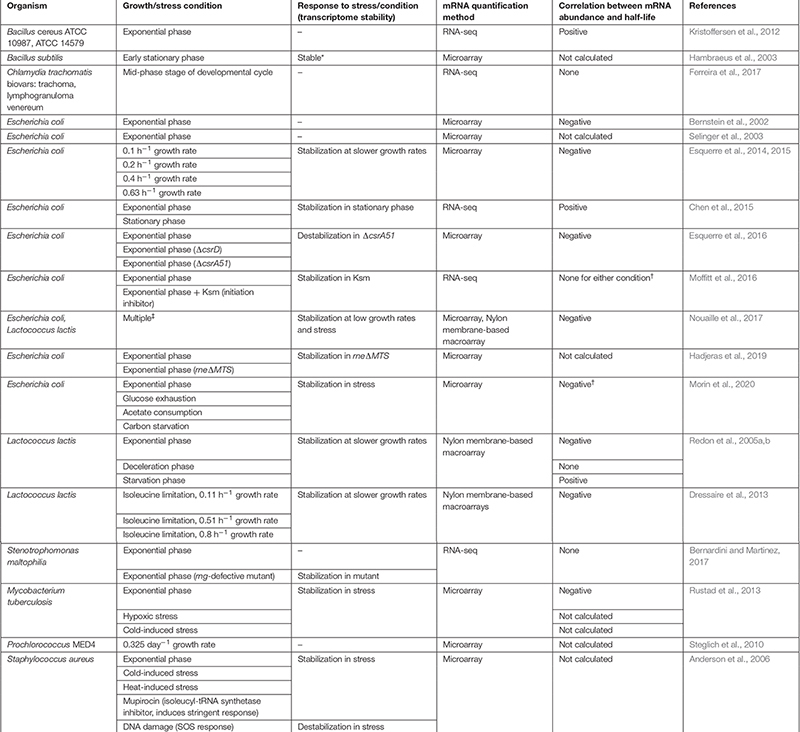

*Not compared to an exponential phase culture within the same study. Stabilization report based on previously reported studies. ^†^Our analysis of the source data. ^‡^Includes data for L. lactis at growth rates of 0.09, 0.24, 0.35, and 0.47 h^–1^ (Dressaire et al., 2013); and unpublished and previously published data for E. coli at growth rates of 0.04, 0.11, 0.38, 0.51, and 0.80 h^–1^ and stationary phase (Esquerre et al., 2014).

In this review, we will discuss a range of reported situations in which bacterial mRNA stability is modulated in response to various stress conditions, with a focus on known and suspected mechanisms underlying such regulation. We will also discuss the ways in which known gene-specific mechanisms shape our thinking on the unanswered question of how mRNA pools are globally stabilized in response to energy stress. Furthermore, we will discuss the ways in which regulation of mRNA stability in clinically relevant bacteria, such as *Mycobacterium tuberculosis*, shape their responses to the host environment.

## RNases and Other Degradation Proteins

### The Degradosome

RNA degradation is carried out by a wide range of RNases, enzymes with strong activities and relatively low specificities toward their targets (reviewed in Carpousis, 2007). There are two main types of RNases: endonucleases and exonucleases. The former cleave RNA sequences at internal points, while the latter carry out nucleolytic attacks from either end of the RNA chain (deemed 5′ or 3′ exonucleases based on their enzymatic directionality). Some bacteria possess both 5′ and 3′ exonucleases – *M. tuberculosis* and *Mycobacterium smegmatis*, for example – while others such as *E. coli* have only 3′ exonucleases.

**FIGURE 2:**
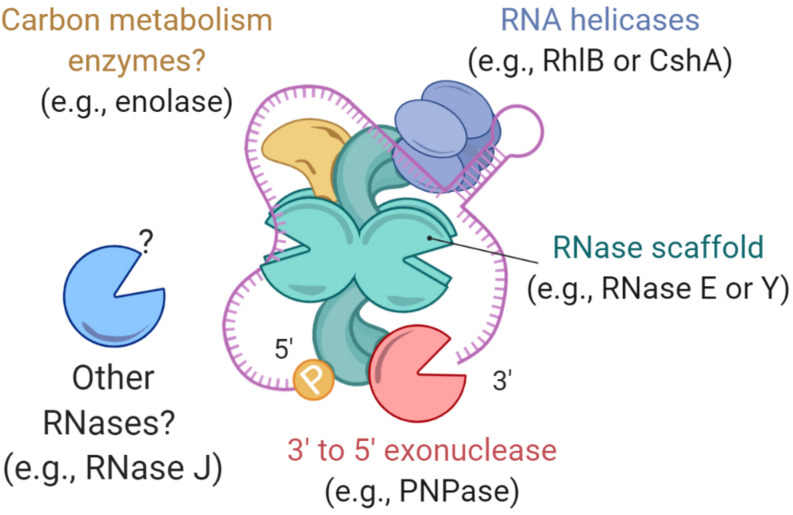
Bacterial degradosomes. The bacterial degradosome is scaffolded by an RNase such as RNase E in *E. coli* and RNase Y in *B. subtilis*. The RNase scaffolds have catalytic domains and natively disordered scaffold domains that bind other degradosome proteins. Typical degradosome components in both gram-positive and gram-negative bacteria are RNA helicases, carbon metabolism enzymes, and other RNases.

With respect to RNA degradation systems, *E. coli* is perhaps the most studied organism. In fact, it was in *E. coli* that a multiprotein complex, deemed the degradosome (Figure 2), was first reported (Carpousis et al., 1994; Py et al., 1994). In *E. coli*, the main degradosome components are two RNases (RNase E and PNPase), a DEAD-box RNA helicase (RhlB), and a glycolytic enzyme (enolase) (Carpousis et al., 1994; Py et al., 1994; Marcaida et al., 2006; Carpousis, 2007). RhlB facilitates RNase activity by unwinding stem-loops within RNA targets (Py et al., 1996). Both RNases carry out RNA degradation (Mohanty and Kushner, 2000; Deutscher, 2006; Unciuleac and Shuman, 2013). Moreover, in this bacterium the C-terminal region of RNase E acts as a scaffold for other degradosome components (Kido et al., 1996; Vanzo et al., 1998; Lopez et al., 1999; Morita et al., 2004). However, not all of the degradosome components are well defined or have known roles. For example, enolase is suspected to have a regulatory role in mRNA degradation under low phosphosugar levels (Morita et al., 2004; Chandran and Luisi, 2006) and anaerobic conditions (Murashko and Lin-Chao, 2017).

While RNases can degrade RNA substrates on their own, it has been suggested that degradosomes increase the efficiency of RNA degradation, for example by facilitating processing of structures such as stem-loops and repeated extragenic palindromic sequences (Newbury et al., 1987; McLaren et al., 1991; Py et al., 1996). Alteration of the degradosome components leads to changes in transcriptome stability; for example, deletion of RhlB in *E. coli* results in longer mRNA half-lives (Bernstein et al., 2004). Similarly, mRNA stability is dramatically increased when the arginine−rich RNA binding region or the scaffolding region of RNase E are deleted (Kido et al., 1996; Ow et al., 2000). While the RNA degradosome of *E. coli* has been extensively studied, the composition and function of degradosomes in other gram-negatives and in gram-positives may differ, and new studies are still uncovering this information. In the Firmicute *Bacillus subtilis*, there is no RNase E homolog. Instead, RNase Y serves as a degradosome scaffold for PNPase, the helicase CshA (Lehnik-Habrink et al., 2010), phosphofructokinase (Commichau et al., 2009), and RNase J1 and RNase J2 – two bifunctional enzymes with both endonucleolytic and 5′–3′ exoribonuclease activity (Even et al., 2005; Shahbabian et al., 2009; Mathy et al., 2010; Durand et al., 2012). Interestingly, the *B. subtilis* degradosome interactions have been shown mainly by bacterial 2-hybrid assays and immunoprecipitation of complexes stabilized by formaldehyde crosslinking (Commichau et al., 2009; Lehnik-Habrink et al., 2010), in contrast to the *E. coli* degradosome which can be immunoprecipitated without a crosslinking agent (Carpousis et al., 1994; Py et al., 1994, 1996). This suggests that *B. subtilis* degradosomes could be more transient in nature. A recent report on the Actinomycete *M. tuberculosis* provided insight into its elusive degradosome structure, which appears to be composed of RhlE (an RNA helicase), PNPase, RNase E, and RNase J (Plocinski et al., 2019). Overall, the degradosome is considered to be the ultimate effector of bulk mRNA degradation in bacterial cells, but it has also been implicated in regulating the stability of specific mRNAs and sRNAs, as will be discussed in later sections. For further details on the degradosome, we encourage reading the following reviews (Carpousis, 2007; Bandyra et al., 2013; Ait-Bara and Carpousis, 2015; Cho, 2017; Tejada-Arranz et al., 2020).

### An Overview of RNase Regulation

There are multiple ways in which transcript levels can be regulated. Alteration of mRNA steady-state abundance is ultimately a consequence of changes in transcription, changes in mRNA half-life, or both. In the process of mRNA degradation, the roles of different RNases may be defined in part by their preferred cleavage sequences. In *Staphylococcus aureus*, RNase Y cleavage is usually in the R↓W sequence, near AU rich regions (Khemici et al., 2015). This pattern seems to be conserved in *B. subtilis* (Shahbabian et al., 2009). Furthermore, in these two gram-positive organisms, RNase Y cleavage appears to be influenced by proximity to a secondary structure. In *E. coli*, RNase E cleaves single-stranded RNA with a strong preference for the +2 sites in RN↓AU (Mackie, 1992; McDowall et al., 1994), or in RN↓WUU in *Salmonella enterica* (Chao et al., 2017). In *M. smegmatis*, a strong preference for cleavage 5′ of cytidines was detected in a transcriptome-wide RNA cleavage analysis (Martini et al., 2019). RNase E could be responsible for these cleavage events, given its major role in mycobacteria, however, we cannot yet exclude the possibility that they are produced by another endonuclease. In contrast, RNase III in *E. coli* has optimal activity on double-stranded RNA, where the cleavage site is specified by both positive and negative sequence and secondary structure determinants (Pertzev and Nicholson, 2006). While the preferred cleavage sites of various RNases seem highly represented in the mRNA pool, some transcripts are more resistant to cleavage than others, indicating the presence of mechanisms that regulate not only bulk RNA stability, but also differential stabilities among transcripts.

**FIGURE 3:**
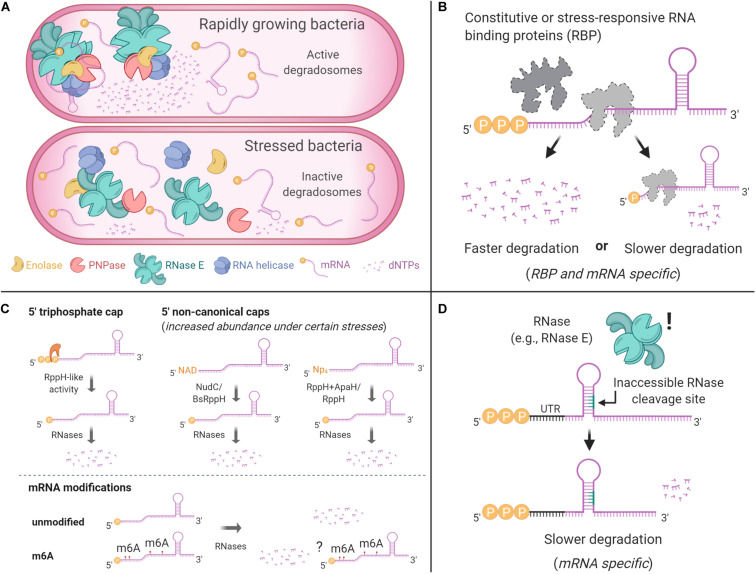
Common mechanisms that can protect mRNAs from degradation. **(A)** Degradosome localization can influence its RNA degradation activity. In *E. coli*, the degradosome is anchored to the cytoplasmic membrane via RNase E’s N-terminal domain, where it displays higher RNA processing activity in degradation foci. A cytoplasmic RNase E is less efficient in degradosome assembly and RNA processing. In *B. subtilis*, RNase Y is associated with the membrane and is more active when in smaller foci and less active when in larger foci. **(B)** RNA binding proteins can modulate mRNA degradation. Some of them, such as CsrA in γ-Proteobacteria, have regulatory roles as a response to environmental changes. **(C)** The chemical nature of mRNA 5′ ends can protect transcripts from degradation. These caps may vary depending on stress conditions. Nucleotide modifications in the bodies of transcripts have also been reported, but they have not been shown to alter mRNA stability. **(D)** RNA degradation depends on RNase accessibility to cleavage sites. Secondary structures that block cleavage sites can result in slower RNA degradation.

**FIGURE 4:**
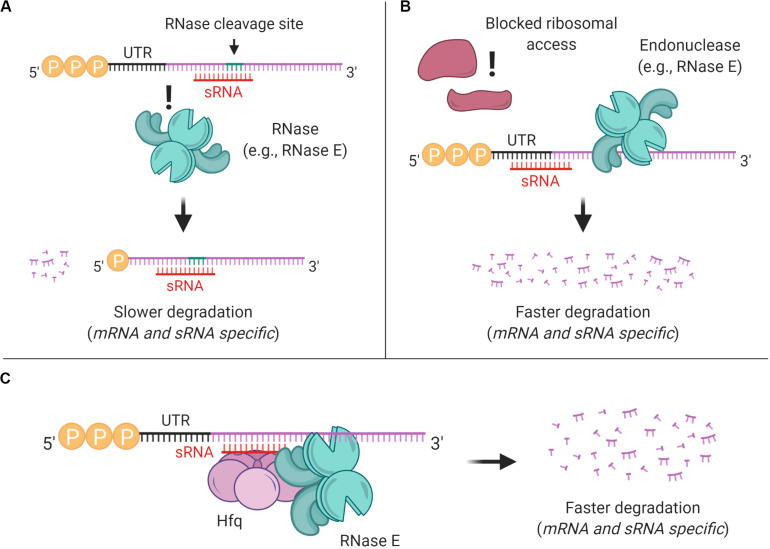
sRNAs can affect mRNA stability through multiple mechanisms. **(A)** sRNA binding can mask preferred RNase cleavage sites, thereby stabilizing transcripts. **(B)** sRNA binding can block ribosome access to Shine-Dalgarno sites, reducing translation and typically destabilizing transcripts. **(C)** In *E. coli* and some other gram-negative bacteria, sRNA-mRNA pairing is often mediated by Hfq, which typically leads to mRNA degradation.

Studies of various mRNAs have identified multiple features that confer protection against RNase cleavage (Figures 3, 4A). These include stem-loops (Emory et al., 1992; McDowall et al., 1995; Arnold et al., 1998; Hambraeus et al., 2002), 5′ UTRs and leader/leaderless status (Chen et al., 1991; Arnold et al., 1998; Unniraman et al., 2002; Nguyen et al., 2020), subcellular compartmentalization (Khemici et al., 2008; Montero Llopis et al., 2010; Murashko et al., 2012; Khemici et al., 2015; Moffitt et al., 2016), 5′ triphosphate groups (Bouvet and Belasco, 1992; Emory et al., 1992; Arnold et al., 1998; Mackie, 1998), 5′ NAD^+^/NADH/dephospho-coenzyme A caps (Chen et al., 2009; Kowtoniuk et al., 2009; Bird et al., 2016; Frindert et al., 2018), Np_n_N caps (Luciano et al., 2019; Hudecek et al., 2020), and association with regulatory proteins and sRNAs (Braun et al., 1998; Gualerzi et al., 2003; Moll et al., 2003; Afonyushkin et al., 2005; Daou-Chabo et al., 2009; Nielsen et al., 2010; Morita and Aiba, 2011; Faner and Feig, 2013; Liang and Deutscher, 2013; Deng et al., 2014; Sinha et al., 2018; Zhao et al., 2018; Cameron et al., 2019; Chen H. et al., 2019; Richards and Belasco, 2019). For example, in *Streptococcus pyogenes* the sRNA FasX binds to the 5′ end of *ska* – a transcript coding for streptokinase – increasing its mRNA half-life, thus allowing an extended period of time in which translation of streptokinase can occur (Ramirez-Pena et al., 2010). In other cases, the product of an mRNA can regulate its own transcript stability. In *E. coli*, the fate of the *lysC* transcript is regulated by a dual-acting riboswitch that, under low levels of lysine, promotes translation initiation while simultaneously sequestering RNase E cleavage sites. In the presence of lysine, the riboswitch folds into an alternative conformation that exposes RNase E cleavage motifs, in addition to blocking translation (Caron et al., 2012). In these examples, it is ultimately the conformational structure of the mRNA that allows regulation of its half-life, independently from the stability of the bulk mRNA pool.

The activity of RNases does not always result in RNA decay. Some mRNA precursors can be processed by RNases to create mature, functional forms of the transcript (Condon et al., 1996). In a similar manner, polycistronic transcripts can be cleaved by endonucleases to produce transcripts with varying degrees of stability; some examples include (Belasco et al., 1985; Baga et al., 1988; Nilsson and Uhlin, 1991; Nilsson et al., 1996; Ludwig et al., 2001; Esquerre et al., 2014; Xu et al., 2015). While this is a fascinating mechanism of gene-specific regulation, it is beyond the scope of this review.

## mRNA Stabilization as a Response to Stress

When bacteria are forced to slow or stop growth in response to stress, they must reduce their rates of protein synthesis. This can be done by direct modulation of translation or by regulation of transcription and transcript degradation rates. In recent decades, there have been many reports of mRNA stabilization as a response to different stressors, usually conditions that alter growth rate (see Table 1). In *E. coli*, the outer membrane protein A precursor transcript, *ompA*, is very stable in rapidly growing cells (Nilsson et al., 1984), but its half-life is significantly decreased in conditions of slow growth rate (Nilsson et al., 1984; Emory et al., 1992; Vytvytska et al., 2000). An inverse phenomenon was observed in stationary phase *E. coli* cells for

*rpoS* and *rmf*, transcripts coding for the transcription factor σ38 and the ribosome modulation factor, respectively (Zgurskaya et al., 1997; Aiso et al., 2005). Research conducted in other organisms also showed regulation of degradation rates of specific mRNAs according to growth rate: *sdh*, coding for succinate dehydrogenase in *B. subtilis*, and *rpoS* in *Salmonella dublin* had mRNA half-lives negatively correlated with growth rate (Melin et al., 1989; Paesold and Krause, 1999). Furthermore, cell growth studies using chemostats revealed that most transcripts in *E. coli* stabilize at low growth rates (Esquerre et al., 2014), with those belonging to the COGs “Coenzyme transport and metabolism” and “Intracellular trafficking, secretion and vesicular transport” being enriched among the most highly stabilized transcripts. On the other hand, genes in “Cell motility” and “Secondary metabolites biosynthesis, transport and catabolism” had shorter half-lives than the transcript population mean (Esquerre et al., 2015). This reinforces the ideas that transcript half-lives may be linked to gene function and can be regulated as conditions require. For example, in *E. coli*, genes from the COGs “Carbohydrate transport and metabolism” and “Nucleotide transport and metabolism” are amongst the most stable at normal growth rates (Esquerre et al., 2014, 2015, 2016). Although these findings propose a link between growth rate and mRNA stability, it is possible that metabolic status rather than growth rate *per se* is the key determinant of global mRNA stability. In *M. smegmatis*, a drug-induced increase in metabolic activity resulted in accelerated mRNA decay and vice versa, even though growth was halted in both conditions (Vargas-Blanco et al., 2019). Another study supported these findings, showing that mRNA stabilization upon changes in nutrient availability could be dissociated from changes in growth rate (Morin et al., 2020).

Growth rate is altered as a consequence of metabolic changes as bacteria adapt to different environments. Because the ultimate goal of an organism is to survive and multiply, we can assume that in stress conditions – such as low-nutrient environments – bacteria trigger mechanisms that regulate energy usage and preserve energetically expensive macromolecules, such as mRNA. Thus, transcript stabilization is a logical response to various forms of energy stress. Indeed, *E. coli* stabilizes most of its transcriptome in anaerobic conditions (Georgellis et al., 1993) as well as in carbon starvation and stationary phase (Esquerre et al., 2014; Chen et al., 2015; Morin et al., 2020). Studies on *Rhizobium leguminosarum*, *Vibrio* sp. *S14*, and *Lactococcus lactis* also showed increased transcriptome half-lives when the bacteria are subjected to nutrient starvation (Albertson et al., 1990; Thorne and Williams, 1997; Redon et al., 2005a,b). *S. aureus* induces global mRNA stabilization in response to low and high temperatures, as well as during the stringent response (Anderson et al., 2006). Under hypoxic conditions, the median mRNA half-life in *M. tuberculosis* increases from ∼9.5 min to more than 30 min, and cells shifted from 37°C to room temperature stabilized their transcriptomes so dramatically that half-lives could not be measured (Rustad et al., 2013). Similarly, transcript stabilization occurs in *M. smegmatis* in response to carbon starvation and hypoxia (Smeulders et al., 1999; Vargas-Blanco et al., 2019). Intriguingly, transcript destabilization can be resumed within seconds upon re-oxygenation of hypoxic *M. smegmatis* cultures, suggesting a highly sensitive mechanism regulating mRNA degradation in response to stress and energy status (Vargas-Blanco et al., 2019).

This response seems to be conserved even in some eukaryotes such as *Saccharomyces cerevisiae*, where the mRNA turnover rate is slower under stress than in log phase (Jona et al., 2000), and in plants as part of their immune response (Yu et al., 2019). However, the adaptive mechanism(s) underlying global mRNA stabilization as a stress response remain unknown. In the following sections we will discuss in more detail diverse bacterial strategies that contribute to global and gene-specific regulation of RNA stability. Our intent is to highlight recent findings on regulation of RNA degradation, to serve as a base for development of experiments to uncover how mRNA stabilization occurs as a response to stress.

### Regulation of RNA Degradation Proteins

In this section we will discuss factors that have been shown to regulate the abundance and activity of endo- and exonucleases. We invite the reader to consult some excellent reviews (Condon, 2003; Arraiano et al., 2010; Bechhofer and Deutscher, 2019) for additional information on the roles and activities of RNases.

As we described in a previous section, RNases have preferred cleavage sequences. These patterns can be either masked or exposed by alternative RNA folding configurations as a result of intracellular changes, allowing modulation of specific cleavage events, e.g., the *lysC* riboswitch which is sensitive to lysine concentration (Caron et al., 2012). However, this regulatory paradigm tends to be used to control specific messages rather than the overall transcriptome stability. Hence, a major open question is: Are there elements that control RNase abundance or RNase activity that regulate transcriptome stability globally?

Abundance of key RNases that catalyze rate-limiting steps in mRNA degradation can affect bulk mRNA decay. For example, depletion or mutation of RNase E caused bulk mRNA stabilization in *E. coli* (Lopez et al., 1999; Sousa et al., 2001); depletion or mutation of RNase Y caused bulk mRNA stabilization in *B. subtilis* and *S. pyogenes* (Shahbabian et al., 2009; Chen et al., 2013); depletion of RNase J caused bulk mRNA stabilization in *Helicobacter pylori* (Redko et al., 2016); and deletion of RNases J1 and J2 caused mRNA stabilization in *B. subtilis* (Even et al., 2005). Mechanisms for regulation of RNase abundance have been reported in some bacteria. In *E. coli*, RNase III autoregulates its abundance by cleaving its own operon to induce its degradation when RNase III protein levels are high (Bardwell et al., 1989; Matsunaga et al., 1996, 1997; Xu et al., 2008). Similarly, in *E. coli* a stem-loop located in the 5′ UTR of *rne* responds to changes in RNase E levels, allowing this enzyme to autoregulate its own production (Diwa et al., 2000; Diwa and Belasco, 2002). There is evidence that in some cases, stability of other mRNAs can be regulated by changes in RNase abundance. In *E. coli*, the *betT* and *proP* transcripts, encoding osmoregulators, showed increased abundance and stability when cells were subject to osmotic stress, apparently as a consequence of lower RNase III concentrations (Sim et al., 2014). However, there is not yet evidence that global stress-induced mRNA stabilization can be attributed to reduced RNase abundance. In *M. tuberculosis*, a quantitative proteomics study comparing exponentially growing and hypoxic cultures showed no alteration in levels of RNase E, RNase J, RNase III, PNPase, or the helicase HelY even after 20 days under hypoxia (Schubert et al., 2015). Only one RNA helicase, RhlE, had reduced levels in hypoxia (Schubert et al., 2015). Similarly, a study of *M. smegmatis* showed no variation in levels of RNase E, PNPase, or the predicted RNA helicase msmeg_1930 under hypoxia, re-aeration, or exponential growth (Vargas-Blanco et al., 2019). Because mycobacterial transcriptomes are rapidly stabilized upon encountering hypoxia and other stress conditions (Rustad et al., 2013; Vargas-Blanco et al., 2019), it is unlikely that alteration of RNase abundance is part of the early RNA stabilization responses in these organisms.

It is possible that the activity of existing RNA degradation enzymes is regulated. RNA helicases are ATP-dependent, and ATP levels decrease in some bacteria in severe energy stress (Rao et al., 2008; Vargas-Blanco et al., 2019). This raises the possibility that RNA degradation could be directly modulated by ATP levels. However, when this hypothesis was tested in *M. smegmatis*, mRNA stabilization was found to occur prior to a decrease in intracellular ATP levels upon exposure to hypoxic conditions (Vargas-Blanco et al., 2019). While these findings suggest that nucleotide sensing – particularly changes in ATP concentrations – does not influence the initial global stabilization response in mycobacteria, it is possible that ATP concentrations or ATP/ADP ratios could be responsible for further stabilization in later stages of dormancy, and/or that ATP levels contribute to global mRNA stabilization in other bacteria. The roles of nucleotides associated with the stringent response are discussed separately below.

In *E. coli*, inhibition of RNase E activity by RraA and RraB (Regulator of ribonuclease activity A and B) result in increased bulk mRNA half-life (Lee et al., 2003). However, in the case of RraA, the effect was observed after a significant overexpression of the inhibitor (Lee et al., 2003), something not observed under stress. Alternatively, inhibition of RNase activity by other factors may regulate transcript degradation. RNase E was recently shown to have a 5′ linear scanning function, and its cleavage activity is impaired upon encountering obstacles, such as sRNAs or ribosomes (Richards and Belasco, 2019). Furthermore, in *E. coli*, the activity of RNase E has been shown to depend on its anchorage to the inner membrane (Figure 3A). YFP-tagged RNase E forms small foci localized at the inner membrane (Strahl et al., 2015) which are dependent on metabolic activity; in anaerobic conditions RNase E rapidly dissociates from the membrane and diffuses in the cytoplasm, a response apparently dependent on enolase (Murashko and Lin-Chao, 2017). A cytoplasmic version of RNase E was unstable, and led to increased mRNA half-lives (Hadjeras et al., 2019). Interestingly, the cytoplasmic RNase E was able to assemble a degradosome and had a comparable *in vitro* activity to wild type RNase E, supporting the role of membrane attachment and cellular localization in RNase E activity (Moffitt et al., 2016; Hadjeras et al., 2019). Conversely, in *Caulobacter crescentus*, RNase E is cytoplasmic and forms bacterial ribonucleoprotein (BR) bodies, which dynamically assemble and disassemble in the presence of mRNA (Al-Husini et al., 2018). BR body formation was dependent on the RNase E scaffold domains and the presence of mRNA, while disassembly of the bodies required mRNA cleavage (Al-Husini et al., 2018). Intriguingly, the formation of BR-bodies increased under some stress conditions but was unaffected by others, suggesting they play an as-yet undefined role in stress response (Al-Husini et al., 2018). Further work is needed to understand the extent to which RNase localization contributes to regulation of mRNA degradation rates in various species.

In *B. subtilis*, the activity of RNase Y appears to be regulated by both subcellular localization and association with proteins termed the Y-complex (YaaT, YlbF, and YmcA). The Y-complex affects expression of genes involved in biofilm formation, sporulation, and competence, and in some cases, this was shown to be a direct consequence of altered mRNA degradation rates for the relevant genes (Tortosa et al., 2000; Carabetta et al., 2013; DeLoughery et al., 2016; Dubnau et al., 2016). The Y complex has been viewed as a specificity factor for RNase Y, required in particular for processing of polycistronic transcripts (DeLoughery et al., 2018). RNase Y also localizes in the cell membrane, where it can form RNase Y foci (Hunt et al., 2006; Lehnik-Habrink et al., 2011; Hamouche et al., 2020). These foci seem to represent a less active form of the enzyme, as they increased in size in absence of RNA or in Y-complex mutants (Hamouche et al., 2020).

### The Stringent Response and mRNA Degradation

The stringent response is perhaps one of the most well-studied mechanisms of prokaryotic stress adaptation. This response is modulated by guanosine-3′,5′-bisphosphate (ppGpp) and/or guanosine-3′-diphosphate-5′-triphosphate (pppGpp), alarmones collectively referred to as (p)ppGpp. In gram-negative bacteria, (p)ppGpp is synthesized by RelA in response to uncharged-tRNAs binding ribosomes, or by SpoT, a (p)ppGpp synthase/hydrolase, during fatty acid starvation (Seyfzadeh et al., 1993; Battesti and Bouveret, 2009). In some gram-positive bacteria, (p)ppGpp is synthesized by a dual RelA/SpoT homolog (Atkinson et al., 2011; Frederix and Downie, 2011; Corrigan et al., 2016). Once produced, (p)ppGpp halts the synthesis of stable RNA (tRNAs and ribosomes) while upregulating stress-associated genes and downregulating those associated with cell growth (Gentry et al., 1993; Chakraburtty and Bibb, 1997; Martinez-Costa et al., 1998; Avarbock et al., 2000; Artsimovitch et al., 2004; Corrigan et al., 2016). Intriguingly, (p)ppGpp was reported to inhibit PNPase in the actinomycetes *Nonomuraea* sp. and *Streptomyces coelicolor* but not in *E. coli* (Gatewood and Jones, 2010; Siculella et al., 2010), suggesting the stringent response may have a previously overlooked role in directly regulating mRNA degradation in some groups of bacteria. However, a recent study on the stringent response in *M. smegmatis* showed that (p)ppGpp was not required for mRNA stabilization in response to carbon starvation or hypoxia (Vargas-Blanco et al., 2019).

In the pathogen *Borrelia burgdorferi*, a connection between the stringent response and the expression of 241 sRNAs was recently stablished, 187 of which were upregulated during nutrient stress (Drecktrah et al., 2018). The authors of the aforementioned study described potential mechanisms of regulation by Rel_Bbu_ on transcription and fate of some transcripts, such as destabilization of the glycerol uptake facilitator transcript, *glpF*. The SR0546 sRNA is among the sRNAs induced by nutrient starvation; the upregulation of its target, *bosR*, encoding a transcriptional regulator, may suggest a regulatory role of (p)ppGpp on specific mRNA stabilization. However, the effects of these stringent response-induced sRNAs on mRNA stability have not yet been directly tested.

A surprising role of RelZ (initially called MS_RHII-RSD), a dual (p)ppGpp synthase and RNase HII, was reported for *M. smegmatis* (Murdeshwar and Chatterji, 2012). R-loops (RNA/DNA hybrids) are harmful structures that cause replication stress and can be removed by the RNase H domain of RelZ, while stalled ribosome removal is attributed to their alarmone synthase domain. RelZ was shown to be upregulated under short UV exposure in *M. smegmatis* (Krishnan et al., 2016), and while its role is suspected to increase cell viability under stress conditions (Petchiappan et al., 2020), the stringent response seems to not intervene in transcriptome stability regulation. This pathway leads to degradation of transcripts involved in R-loops, but given the low frequency of R-loop formation, the effects on mRNA pools are likely to be minimal.

Overall, there is much evidence that the stringent response regulates expression of specific transcripts in various bacteria. However, the extent to which control of mRNA stability contributes to these effects is mostly untested. The stringent response also plays important roles in mediating global responses to starvation and other forms of energy stress, but there is not yet evidence that it contributes to global mRNA stabilization, which is a consistent component of these stress responses. This suggests that the stringent response may not be the mediator of global mRNA stabilization in response to stress, or that its involvement in this process is species-specific.

### Transcript Modifications as Regulators of mRNA Decay

Bacterial mRNA is primarily transcribed using nucleoside triphosphates as initiating nucleotides, making mRNAs triphosphorylated at their 5′ ends. In *S. aureus*, RNase J1 exhibits strong *in vitro* exo- and endonucleolytic activities on 5′ triphosphorylated transcripts (Hausmann et al., 2017). However, in most other organisms studied to date, RNases E, J, and Y more efficiently cleave mRNAs with 5′ monophosphates (Figure 3C). RNase E is an endoribonuclease, but has a binding pocket for monophosphorylated 5′ ends (Callaghan et al., 2005) that strongly stimulates its activity in organisms including *E. coli* and *M. tuberculosis* (Mackie, 1998; Zeller et al., 2007). Similarly, in *B. subtilis*, RNase J1, and to a lesser extent J2, show a strong preference toward 5′ monophosphorylated substrates (Even et al., 2005). RNase Y also shows preference toward monophosphorylated 5′ substrates, but to a lesser extent (Shahbabian et al., 2009). These findings contributed to the discovery of RppH, an RNA pyrophosphohydrolase. Similar enzymes were later found in other bacteria, such as *Bdellovibrio bacteriovorus* (Messing et al., 2009) and *B. subtilis* (Richards et al., 2011). However, while the role of 5′ triphosphate pyrophosphohydrolysis was initially attributed to RppH (Celesnik et al., 2007; Deana et al., 2008), recent findings have shown that the primary substrate of RppH in *E. coli* is 5′ diphosphorylated RNAs, and that 5′ diphosphorylated RNAs are abundant in the transcriptome (Luciano et al., 2017). As RppH cannot convert 5′ triphosphates to diphosphates, this suggests the existence of an unknown 5′ triphosphate to diphosphate phosphorylase. Given that 5′ monophosphates make transcripts more susceptible to degradation in multiple organisms, one could envision regulation of 5′ triphosphate pyrophosphohydrolysis as a potential mechanism for regulation of mRNA stability. However, to our knowledge there are not yet reports of if and how pyrophosphohydrolysis or γ-phosphate removal are regulated.

The presence of non-canonical mRNA 5′ ends has recently been reported for subsets of mRNAs in several bacterial species, suggesting another possible mechanism for regulation of mRNA stability (Figure 3C). Examples include NADH and NAD+ (Chen et al., 2009; Cahova et al., 2015), and less commonly, dephospho-CoA, succinyl-CoA, acetyl-CoA, and methylmalonyl-CoA (Kowtoniuk et al., 2009). We will refer to these as 5′ caps, with the understanding that they are structurally and functionally distinct from eukaryotic mRNA caps. Other studies have shown additional types of 5′ capping, as well as potential mechanisms behind it (Bird et al., 2016; Zhang et al., 2016; Julius and Yuzenkova, 2017). In most cases, bacterial caps are incorporated directly into mRNAs during transcription initiation. RNA polymerase can initiate transcription with non-canonical nucleotides such as NAD in *E. coli* (Bird et al., 2016; Vvedenskaya et al., 2018) and *B. subtilis* (Frindert et al., 2018). Furthermore, *E. coli* RNA polymerase seems to initiate with dinucleoside tetraphosphates (Np_4_N), Np_4_A in particular, with an efficiency almost 60 times higher than for NAD (Luciano and Belasco, 2020). Alternative, posttranscriptional mechanisms may also contribute to Np_4_ capping formation, as *in vitro* experiments using LysU (lysyl-tRNA synthetase) from *E. coli* suggest (Luciano et al., 2019).

The intracellular concentration of Np_4_As were shown to be affected by overproduction of aminoacyl-tRNA synthetases (Brevet et al., 1989). Interestingly, some stress conditions also induce higher levels of Np_4_Ns, for example heat shock (Lee et al., 1983), oxidative stress (Bochner et al., 1984), cadmium stress (Coste et al., 1987; Luciano et al., 2019) and disulfide stress (Bochner et al., 1984; Luciano et al., 2019). 5′ mRNA decapping was shown to require Nudix enzymes, such as NudC and BsRppH, to hydrolyze NAD-RNA substrates (Hofer et al., 2016; Frindert et al., 2018). On the other hand, hydrolysis of Np_4_As requires RppH and ApaH, the latter carrying out the hydrolysis of Np_4_As into two NDPs (Farr et al., 1989); in this context ApaH generates a diphosphorylated 5′ end that can be readily converted to monophosphate 5′ end by RppH (Figure 3C). Non-canonical mRNA 5′ ends also occur when transcription initiates with short RNA degradation products, resulting in mRNAs with 5′ hydroxyls (Druzhinin et al., 2015). Such transcripts have been found in *E. coli* and *Vibrio cholerae* and are present at increased abundance in stationary phase (Vvedenskaya et al., 2012; Druzhinin et al., 2015). However, the effects of these alternate 5′ ends on transcript stability have not been reported.

Some mRNA caps have been shown to stabilize mRNAs in *E. coli* (Bird et al., 2016; Luciano et al., 2019) and in *B. subtilis* (Frindert et al., 2018). For example, after increasing the cellular concentration of Np_4_Ns in cadmium-stressed cells and in Δ*apaH* mutants, RNA stability was increased, suggesting that Np_4_ caps have a stabilizing role (Luciano et al., 2019). Additionally, in this study Np_4_ caps were suggested to be more abundant than NAD caps. Similarly, in the *E. coli* Δ*nudC* mutant strain there is an increase of up to fourfold in RNA stability for transcripts with non-canonical 5′ caps (Bird et al., 2016). Furthermore, NAD 5′ caps were almost twofold more abundant for cells in stationary phase when compared to exponential phase (Bird et al., 2016). Together, these findings present a potential mechanism for stabilization of mRNA under stress conditions. An interesting regulatory mechanism behind Np_4_ decapping in *E. coli* was recently linked to methylation in m^7^Gp_4_Gm and m^6^Ap_3_A 5′ caps, which protects them from RppH cleavage but not from AppH (Hudecek et al., 2020). Methylated Np_n_N caps were shown to be more abundant in stationary phase than exponential phase (Hudecek et al., 2020), consistent with the idea that these caps protect mRNA from degradation. Interestingly, the Np_n_N caps found in that study did not include Ap_4_N (Hudecek et al., 2020), presumably due to different stress conditions and detection techniques than those in Luciano et al. (2019). Since capped mRNAs appear to be generally more stable than canonical mRNAs, it is logical to infer that when stress conditions cause growth to slow or stop and transcription to slow or stop concomitantly, the proportion of capped mRNAs will increase as a result of their inherently longer half-lives. One could therefore speculate that the global mRNA stabilization observed in non-growing bacteria is due in part to an mRNA pool that is largely protected by 5′ caps. This is plausible assuming capping frequency remains constant or increases under stress. But, a recent study argues against this idea. Rapid transcript destabilization occurred in hypoxic *M. smegmatis* cultures after re-exposure to oxygen, even when transcription was blocked prior to re-aeration (Vargas-Blanco et al., 2019). Thus, mRNA capping does not explain the transcript stabilization observed in these conditions (early-stage hypoxia) – at least in *M. smegmatis* – but could be involved in mRNA stabilization in other conditions and/or other bacteria.

Another possible mechanism of mRNA stabilization involves posttranscriptional nucleotide modifications (Figure 3C). *N*^6^-methyladenosine (m^6^A) is a common base modification in mice and humans (Meyer et al., 2012; Linder et al., 2015). This methylation is enriched near stop codons and in 3′ UTRs (Yue et al., 2018), and is dependent on the consensus motif DRACH (Linder et al., 2015). Recent studies revealed m^6^A to be an important part of a transcript stability regulatory mechanism, as it facilitates mRNA degradation in association with RBP in mice, zebra fish, and human cells (Schwartz et al., 2014; Wang et al., 2014; Zhao et al., 2017). Moreover, the levels of m^6^A methylation are responsive to stress conditions, as shown for human cancer cells under hypoxic conditions (Panneerdoss et al., 2018), suggesting a posttranscriptional regulatory role. In *E. coli* and *Pseudomonas aeruginosa*, m^6^A is present at similar levels, ∼0.2–0.3% of adenines (Deng W. et al., 2015), to those reported for yeast and other eukaryotes (Wei et al., 1975; Bodi et al., 2010). However, in contrast to mammals, m^6^A appears distributed throughout the gene, with modest enrichments near the 5′ ends and centers of transcripts, and with a similar m^6^A motif for *E. coli* and *P. aeruginosa* (UGCCAG and GGYCAG, respectively) (Deng X. et al., 2015). Contrary to eukaryotes, m^6^A methylation has not been shown to have a global role in mRNA degradation in bacterial stress responses. A deep analysis in *E. coli* and *P. aeruginosa* revealed no difference in the m^6^A levels for cells growing in LB when compared to other (unspecified) growth media, or oxidative stress; interestingly, increasing the temperature from 37 to 45°C lowered m^6^A methylation levels, but only for *P. aeruginosa* (Deng X. et al., 2015). Furthermore, the m^6^A levels were lower in other bacteria (∼0.02–0.08%, for *S. aureus*, *B. subtilis*, *Anabaena* sp., and *Synechocystis* sp.) (Deng X. et al., 2015), suggesting that this particular base modification may not be conserved across bacteria. In *E. coli*, codon modifications of the *ermCL* mRNA with m^6^A blocked translation, though it had no impact on mRNA degradation rates (Hoernes et al., 2016). While it is conceivable that m^6^A has a role in the regulation of bacterial translation, current evidence does not suggest it regulates mRNA fate.

5-methylcytosine (m^5^C) has also been found in mRNA. In eukaryotes, m^5^C has been shown to increase transcript stability (Arango et al., 2018; Chen X. et al., 2019; Yang et al., 2019; Schumann et al., 2020), while reports on translation regulation are controversial (Huang et al., 2019; Yang et al., 2019; Schumann et al., 2020). m^5^C modifications have been found in mRNA and 23S rRNA in the archaeon *Solfolobus solfataricus* (Edelheit et al., 2013). However, there is no defined role of m^5^C in *S. solfataricus*, and evidence of m^5^C in bacteria or regulatory roles in RNA degradation have not been reported.

Another modification, and perhaps the most abundant in RNA, is pseudouridine (Ψ) (Rozenski et al., 1999). Ψ is present at the position U55 in all *E. coli* tRNAs (Gutgsell et al., 2000), and is widespread across kingdoms (Nishikura and De Robertis, 1981; Becker et al., 1997; Ishida et al., 2011). In *E. coli*, deletion of *truB*, encoding a tRNA Ψ 55 synthase (Nurse et al., 1995), was shown reduce viability after a temperature shock (37–50°C); however, no viability changes were observed during exponential growth at 37°C (Kinghorn et al., 2002). In *Thermus thermophilus*, a Δ*truB* mutant showed a growth defect when cultured at 50°C (Ishida et al., 2011). Thus, it is possible that the presence of tRNA modifications under stress conditions contributes to survival in other bacteria. Other tRNA modifications have been also reported in bacteria and yeast during stress, contributing to a translational bias with implications for translation regulation (Chan et al., 2010, 2012; Laxman et al., 2013; Deng W. et al., 2015; Chionh et al., 2016). However, while stress may alter tRNA modifications, ultimately these changes lead to translational regulation without clear evidence, at least in bacteria, of effects on mRNAs. On the other hand, Ψ modifications on mRNA have been shown to increase mRNA stability in yeast and human cells (Carlile et al., 2014) and in *Toxoplasma gondii* (Nakamoto et al., 2017). A broad study involving *E. coli* and human cells found that even a single replacement of U with Ψ in mRNA can interfere with translation (Eyler et al., 2019). Whether these modifications ultimately regulate mRNA stability in bacteria as a response to stress is an open question. Based on evidence aforementioned for *M. smegmatis* regarding the rapidity of transcript destabilization after stress alleviation (Vargas-Blanco et al., 2019), we speculate that base modifications are unlikely to be the primary mechanism of mRNA stabilization in hypoxic mycobacteria, although it could play roles in other organisms or conditions.

### Roles of Ribosomes, Translation, sRNAs, and RNA-Binding Proteins in Regulation of mRNA Decay

**FIGURE 5:**
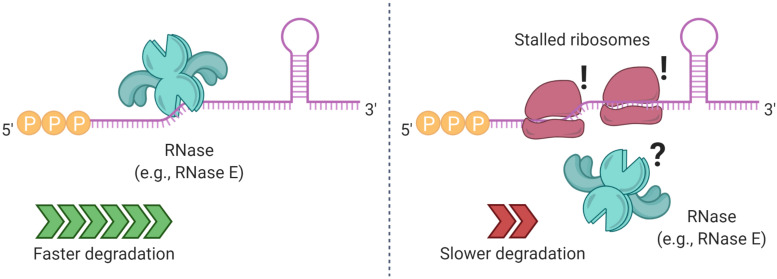
Ribosome binding and stalling can alter mRNA degradation. In some cases, ribosome stalling can mask RNase cleavage sites, increasing the half-life of a transcript. Elements that prevent ribosome binding, such as translation initiation inhibitors, lead to shorter mRNA half-lives.

Experiments conducted by Bechhofer and others in *B. subtilis* showed that ribosome stalling can increase *ermC* half-life. In this scenario, ribosomes acted as obstacles at the 5′ ends of transcripts, resulting in protection from endonucleolytic cleavage downstream (Shivakumar et al., 1980; Bechhofer and Dubnau, 1987; Bechhofer and Zen, 1989). These findings would become early evidence of a 5′–3′ polarity for endonucleolytic activity, dependent upon or enhanced by (1) interaction with a 5′ monophosphate, and (2) RNase linear scanning mechanisms, as it would be later reported by others (Bouvet and Belasco, 1992; Jourdan and McDowall, 2008; Kime et al., 2010; Richards and Belasco, 2016, 2019). In *E. coli*, the use of puromycin or kasugamycin – translation inhibitors that cause ribosomes to dissociate from transcripts – caused faster mRNA decay in the absence of new transcription (Varmus et al., 1971; Pato et al., 1973; Schneider et al., 1978). On the other hand, the use of chloramphenicol, fusidic acid or tetracycline – elongation inhibitors that cause ribosomes to stall on transcripts – resulted in transcript stabilization (Varmus et al., 1971; Fry et al., 1972; Pato et al., 1973; Schneider et al., 1978), findings also later shown in *M. smegmatis* (Vargas-Blanco et al., 2019). These results are consistent with ribosome binding having a protective effect on mRNAs (Figure 5). In experiments where transcription was not blocked, it is possible that the mRNA stabilization seen in response to elongation inhibitors may also be conferred in part by the sudden increase in rRNA synthesis that these drugs cause, which increases the abundance of potential RNase substrates and could therefore titrate the activity of RNases such as PNPase and RNase E (Lopez et al., 1998). However, the increase in rRNA synthesis cannot fully explain these effects.

In *B. subtilis*, the stability of *gsiB*, encoding general stress protein, and *ermC*, encoding erythromycin resistance leader peptide, are associated with ribosome binding (Sandler and Weisblum, 1989; Hambraeus et al., 2000). Mutations to the RBS sites of *gsiB*, *aprE* (coding for subtilisin), and SP82 phage mRNA resulted in reductions of their mRNA half-lives (Hue et al., 1995; Jurgen et al., 1998; Hambraeus et al., 2002). Transcript stability conferred by ribosomes does not always require productive translation, at least for *ermC* (Hambraeus et al., 2002) and *ompA* (Emory and Belasco, 1990), where transcripts were stable in the absence of start codons as long as strong Shine-Dalgarno (SD) sequences were present (Arnold et al., 1998). A later study also in *E. coli* reported that ribosome protection is independent of translation for another transcript (Wagner et al., 1994). Transcript stabilization in a translation-independent manner was also shown for *B. subtilis*, with the insertion of an alternative SD (not involved in translation) to the gene reporter *cryIII* (Agaisse and Lereclus, 1996). These findings suggest that binding of a 30S subunit to a transcript, regardless of translation, may suffice to impair RNase degradation.

However, other studies did find a correlation between translation itself and stability. In *E. coli*, codon composition can influence translation rate and mRNA stability; codon-optimized transcripts were more stable than their corresponding non-modified, inefficiently-translated versions (Boel et al., 2016). Similar results were shown for *S. cerevisiae* (Presnyak et al., 2015). A transcriptome-wide analysis in *E. coli* also identified a positive correlation between mRNA stability and codon content optimality, for bacteria growing at different rates (Esquerre et al., 2015). This directly contradicted a previous report that codon optimality and half-life were inversely correlated (Lenz et al., 2011), possibly due to use of different codon optimality metrics. In *B. subtilis*, translation initiation is necessary to prevent swift degradation of the *hbs* transcript, which encodes the DNA binding protein HBsu (Daou-Chabo et al., 2009; Braun et al., 2017). In *M. smegmatis* and *M. tuberculosis*, RNase E cleaves the *furA-katG* operon, producing an unstable *furA* message that is rapidly degraded while the *katG* transcript is stabilized as it becomes readily accessible for translation (Sala et al., 2008). Overall, regulation of mRNA stability by translation initiation and SD strength seems to be gene-specific.

While it is generally accepted in *E. coli* that occlusion of RNase cleavage sites by ribosome occupancy may protect a transcript from degradation (Joyce and Dreyfus, 1998), ribosome association with mRNA has not been shown to regulate mRNA stability globally in response to stress. However, data from *B. subtilis* suggest an interesting mechanism by which RNase activity could affect translation and therefore mRNA degradation on a transcriptome-wide scale (Bruscella et al., 2011). The *infC-rpmI-rplT* operon, which encodes translation initiation factor 3 (IF-3) along with two ribosomal proteins, is expressed from two promoters. The resulting transcripts have different sensitivities to RNase Y, and the RNase Y-sensitive transcript is not competent for translation of IF-3. As a result, inhibition of RNase Y expression alters the relative abundance of the two transcript and causes reduced translation of IF-3. If this were to cause globally reduced translation due to IF-3 deficiency, mRNA decay could be globally increased as a result, although this effect would presumably be counteracted by the globally reduced RNase Y activity. Complex interplays between RNase levels and translation may therefore have the potential to globally impact mRNA decay in *B. subtilis*.

RNA-binding proteins (RBPs), stalled ribosomes, and SD-like sequences in close proximity to transcript 5′ ends can also alter mRNA fate (Sharp and Bechhofer, 2005). In *B. subtilis*, interaction of the RBP Glp with the 5′ UTR of *glpD*, encoding glycerol-3-phosphate dehydrogenase, increases the transcript’s stability (Glatz et al., 1996). Other RBPs can modulate the stability of target genes during stress conditions (Figure 3B). For example, H-NS, a histone-like protein, regulates the RNA stability of *rpoS* in *E. coli* and *V. cholerae* in stressful environments (Brescia et al., 2004; Silva et al., 2008; Wang et al., 2012). The carbon storage regulator CsrA is an RBP that regulates gene expression posttranscriptionally in *E. coli* and other γ-Proteobacteria in response to environmental changes, described in Timmermans and Van Melderen (2010) and Romeo and Babitzke (2018). CsrA regulatory roles are best studied in *E. coli*. The *glgCAP* transcript, encoding genes implicated in the biosynthesis of glycogen, is destabilized when bound by CsrA (Liu et al., 1995). This response is halted when *E. coli* enters stationary phase, where CsrA is sequestered by the sRNA CsrB in a ribonucleoprotein complex (Liu et al., 1997). Conversely, CsrA was shown to stabilize some transcripts. CsrA directly binds the *pgaA* transcript, increasing its half-life along with the rest of the *pgaABC* polycistron, encoding genes associated to biofilm formation (Wang et al., 2005). Similarly, CsrA stabilizes the *flhDC* transcript, encoding the flagellar activation genes FlhD_2_C_2_ (Wei et al., 2001). More recently, a transcriptome-wide study together with bioinformatics predictions showed a major role for CsrA as an mRNA stabilization factor in *E. coli* (M9 minimal media, doubling time of 6.9 h) for more than a thousand transcripts, of which many were predicted to have at least one putative CsrA binding site (Esquerre et al., 2016). CsrA could directly bind transcripts and protect them from RNases, or could affect mRNA stability indirectly by modulating expression or activity of other post-transcriptional regulators, e.g., the RNA chaperone Hfq, encoded by *hfq*. In *E. coli*, CsrA can bind the *hfq* mRNA at a single binding site that overlaps its SD region, preventing ribosome access and decreasing its half-life, however, in stationary phase CsrA is sequestered, allowing higher expression of Hfq (Baker et al., 2007). Regulatory roles for CsrA in gram-positive bacteria have only recently been reported. In *B. subtilis*, CsrA mediates the interaction of the sRNA SR1 and the *ahrC* mRNA, encoding a transcription regulator of arginine metabolism, to regulate the expression of the arginine catabolic operons (Muller et al., 2019). However, CsrA-SR1 only mildly increased *ahrC* half-life, and it had no impact on SR1 degradation, indicating that the regulation was primarily at the level of protein synthesis (Muller et al., 2019).

The homohexameric Hfq, highly studied in *E. coli* and present in a large number of bacteria (Sun et al., 2002), is an important regulator of mRNA-sRNA pairing. The multiple roles of Hfq include modulation of sRNA-mediated translation blockage or promotion, and regulation of transcript degradation as a direct consequence of altered translation or through translation-independent mechanisms. For example, guiding a cognate sRNA to the 5′ region of mRNAs can result either in translation disruption by preventing the 30S subunit from binding (Figure 4B), or the opposite outcome by disruption of stem-loops that inhibit its binding (Wassarman et al., 2001; Arluison et al., 2002; Moller et al., 2002; Schumacher et al., 2002; Zhang et al., 2003; Afonyushkin et al., 2005; Sittka et al., 2008). Hfq can also allow RNase E access to specific mRNAs, or modulate the synthesis of Poly(A) tails, assisting PNPase in 3′–5′ degradation, as it will be discussed shortly. The physical properties, sequence specificity, protein interaction partners, sRNAs/mRNAs binding kinetics, and other important aspects of Hfq function will not be described here, as they are well described elsewhere; we refer the reader to the following detailed reviews (Vogel and Luisi, 2011; Updegrove et al., 2016; Kavita et al., 2018; Santiago-Frangos and Woodson, 2018).

A common outcome of Hfq sRNA/mRNA interactions is specific regulation of mRNA half-life (Figure 4C). For example, the destabilization of *ptsG*, encoding a glucose permease, in *E. coli* is mediated by the sRNA SgrS as a response to phosphosugar accumulation (Vanderpool and Gottesman, 2004). Similarly, degradation of *ompA* was also shown to be impacted by the specific binding of the sRNA MicA to its translational start site, blocking binding of the 30S ribosomal subunit and recruiting Hfq to promote RNase E cleavage (Lundberg et al., 1990; Vytvytska et al., 2000; Udekwu et al., 2005). While the regulatory roles of Hfq are widely accepted for other gram-negative bacteria as well (Sonnleitner et al., 2006; Cui et al., 2013), in gram-positive bacteria Hfq is less well characterized. Hfq rescue experiments in *E. coli* and *S. enterica* serovar Typhimurium using Hfq from *B. subtilis* and *S. aureus*, respectively, failed at rescuing the phenotypes (Vecerek et al., 2008; Rochat et al., 2012). These findings suggest important structural and/or functional differences in Hfq across evolutionarily divergent groups of bacteria. A study in *B. subtilis* found that the absence of Hfq does not impair growth under almost 2000 conditions including different carbon, nitrogen, phosphorus and sulfur sources, osmolarity or pH changes in a large phenotypic analysis (Rochat et al., 2015). Similar findings were shown for *S. aureus* (Bohn et al., 2007). However, Hfq became necessary for survival in stationary phase (Hammerle et al., 2014; Rochat et al., 2015). Surprisingly, the absence of Hfq in rich media conditions did not alter the transcriptome of *B. subtilis* (Rochat et al., 2015), while in minimal media, 68 mRNAs and a single sRNA were affected (Hammerle et al., 2014). Both of these studies reported transcriptome changes in the absence of Hfq for *B. subtilis* in stationary phase, particularly for sporulation and TA systems. Nevertheless, these changes do not necessarily confer fitness or increased survival (Rochat et al., 2015). Overall, while Hfq was shown to impact the *B. subtilis* transcriptome under certain stress conditions, its role as a regulator of transcript stability seems to greatly vary across species. In another gram-positive, the pathogen *Listeria monocytogenes*, Hfq interacts with the sRNA LhrA, increasing its stability and controlling the fate of its target mRNAs. But, ∼50 other sRNA seem to function in an Hfq-independent manner (Christiansen et al., 2006; Nielsen et al., 2010; Nielsen et al., 2011). Unexpectedly, hypoxia, stationary phase and low temperature (30°C) did not affect sRNA levels in a Δ*hfq* strain (Toledo-Arana et al., 2009). Hence, it seems that Hfq may have a smaller role in control of mRNA stability, and an overall restricted role in sRNA/mRNA regulation in gram-positive bacteria; and it appears to not be required at all in some bacteria, such as mycobacteria, that lack identified Hfq orthologs (Sun et al., 2002).

### mRNA Folding Alters mRNA Decay

**FIGURE 6:**
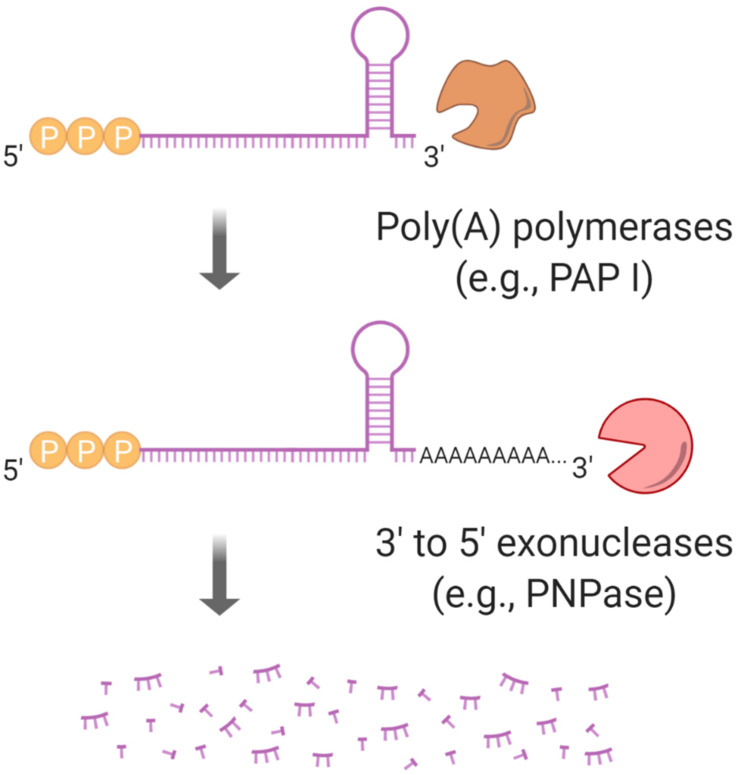
Polyadenylation regulates mRNA half-life. Stem-loops at mRNA 3′ ends block 3′–5′ exoribonucleases such as PNPase. PAP I, a poly(A) polymerase, can facilitate an exoribonuclease “grip” by synthesizing a poly(A) tail.

mRNA secondary structures can modulate translation and transcript stability (Figure 3D). Previously, we have discussed how specific 5′ UTR folding prevents RNase and ribosome accessibility to the *lysC* transcript (Caron et al., 2012). In other transcripts, secondary structures can also prevent RNase E from carrying out the first endonucleolytic cleavage, delaying subsequent steps in the decay pathways. In *Rhodobacter capsulatus*, formation of multiple hairpins can prevent endonucleolytic cleavage of the *puf* operon (Klug and Cohen, 1990). A stem-loop at the 5′ UTR confers stability to *recA*, coding for the nucleoprotein filament RecA in *Acinetobacter baumannii* (Ching et al., 2017), as well as *vacA*, coding for vacuolating cytotoxin A in *Helicobacter pylori* (Amilon et al., 2015). In the case of *vacA*, the stem-loop is also essential for transcript stabilization in acidic and osmotic stress (Amilon et al., 2015). The distance between the start codon and secondary structures can also affect mRNA half-life, as was shown for the ΔermC mRNA in *B. subtilis*, where placing a stem-loop too close to the SD decreased transcript stability (Sharp and Bechhofer, 2005). Secondary structure at transcript 3′ ends also affects stability. The mRNA 3′ end hairpins formed by Rho-independent transcriptional terminators typically stabilize transcripts, as 3′–5′ RNases have difficulty initiating decay without a single-stranded substrate (Adhya et al., 1979; Farnham and Platt, 1981; Abe and Aiba, 1996). In *E. coli*, the poly(A) polymerase (PAP I) is an enzyme responsible for synthesizing poly(A) tails in mRNA (Li et al., 1998). The addition of poly(A) tails to bacterial mRNAs facilitates degradation of transcripts with 3′ hairpins, allowing PNPase – an enzyme that also has a minor polyadenylation role – and other enzymes to carry out exonucleolytic activity (Donovan and Kushner, 1986; Blum et al., 1999; Figure 6).

Thus, it is possible for poly(A) tails to act as regulators of mRNA stability, making PAP I a promising candidate for posttranscriptional regulation. However, while this enzyme has been characterized in *E. coli*, PAP I homologs in *B. subtilis* have not yet been identified (Campos-Guillen et al., 2005). An interesting role of Hfq in *E. coli* was reported for transcripts carrying long poly(A) tails, as binding to the tail prevents the access of PNPase, thereby increasing mRNA stability (Hajnsdorf and Regnier, 2000; Folichon et al., 2005). However, on shorter poly(A) tails (<10 nt), Hfq has poor accessibility, making the transcripts susceptible to the activity of PNPase and RNase II (Regnier and Hajnsdorf, 2013). Interestingly, in *E. coli*, the absence of PAP I disrupts the regulatory role of some sRNAs, leading to an unexpected destabilization of some sRNAs and transcripts, e.g., RyhB and MicA (Sinha et al., 2018). This appears to result from accumulation of transcripts that are normally degraded in a PAP I-dependent fashion. The accumulated transcripts participate in non-specific interactions with sRNAs, leading to degradation of the sRNA-mRNA pairs. Thus, it is suggested that many PAP I targets are transcripts that do not normally interact with sRNAs (Cameron et al., 2019).

Regulation of PNPase abundance has been shown for *E. coli*, as its transcript *pnp* is post-transcriptionally regulated by its own product and RNase III. This mechanism can be disrupted by transcript association with the ribosomal protein S1 (Briani et al., 2008; Carzaniga et al., 2015). Moreover, an increase of the pool of polyadenylated transcripts increases *pnp* half-life, an effect attributed to PNPase titration (Mohanty and Kushner, 2000, 2002). Regardless of this autoregulatory characteristic, changes in PNPase abundance were not detected as a response to hypoxic stress in *M. smegmatis* (Vargas-Blanco et al., 2019), despite increased mRNA stability. While these findings suggest that regulation by mRNA polyadenylation via PNPase abundance is not a mechanism of transcriptome stabilization in mycobacteria, it is possible that polyadenylation activity by other enzymes, such as PcnA and PcnB, Adilakshmi et al. (2000) might have a role in regulation of mRNA turnover in stress. Further research is needed to investigate this possibility.

### The Relationship Between mRNA Abundance and mRNA Decay Rates

In bacteria, the steady-state mRNA concentration is a function of transcription rates and transcript degradation rates, and to a lesser extent, of mRNA dilution. The contribution of mRNA dilution occurring during cell growth is usually ignored, given that doubling times are significantly longer than the median mRNA half-life. For example, in *L. lactis* mRNA half-lives complied with this assumption for 85% of the measured transcripts, at multiple growth rates (Dressaire et al., 2013). In stress conditions, bacterial growth is generally impaired, making the impact of mRNA dilution even smaller and reinforcing the roles of transcription and RNA turnover as the major determinants of mRNA abundance. Also under stress conditions, transcript abundance per cell is typically lower than in conditions of rapid growth. For example, low transcript abundance was observed for *S. aureus* in cold shock, heat shock, and stringent response when compared to unstressed exponential phase (Anderson et al., 2006). The per-cell mRNA concentration decreased in *L. lactis* during progressive adaptation to carbon starvation (Redon et al., 2005a) or isoleucine starvation (Dressaire et al., 2013). The mRNA concentration was three times higher for *E. coli* growing in LB when compared to growth in in minimal media (Bartholomaus et al., 2016). For *M. smegmatis* in early hypoxic stress, the levels of *atpB*, *atpE*, *rnj*, *rraA*, and *sigA* ranged between ∼5 and 75% of those in cells growing in aerobic conditions, and after extended periods of hypoxic or carbon starvation stress, mRNA levels dropped to under 5% of those in log phase (Vargas-Blanco et al., 2019). Given the generally longer half-lives of mRNAs in stressed bacteria, the observation of reduced mRNA concentrations in these conditions may seem counter-intuitive. However, these observations can be reconciled if transcription is also greatly reduced. It is possible that maintaining lower overall mRNA abundance in stress conditions is an adaptive mechanism to favor translation of genes needed for survival of that particular stressor. For example, in a transcriptome-wide study in *E. coli*, mRNA abundance decreased in response to osmotic stress (from ∼2,400 to 1,600 transcripts per cell), a change that may allow specific transcripts – associated with stress response – to be more accessible to ribosomes and translated (Bartholomaus et al., 2016). Interestingly, transcripts with higher copy numbers per cell in normal conditions (>2 copies/cell) were downregulated the most in osmotic stress (Bartholomaus et al., 2016).

**FIGURE 7:**
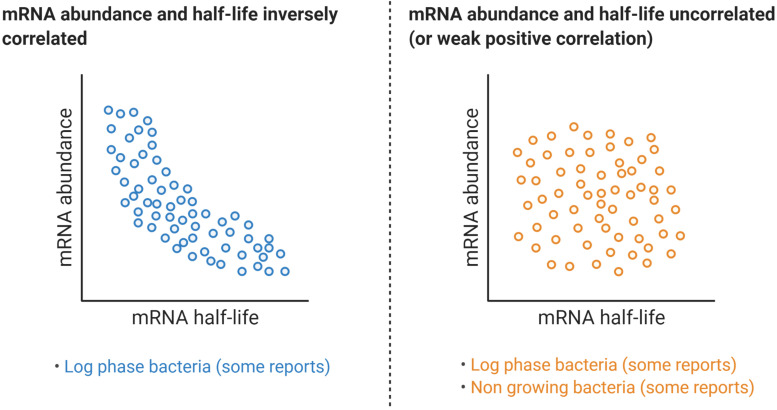
Relationships between mRNA abundance and mRNA decay rates. While some reports have shown a clear negative correlation between a transcript half-life and its abundance, a similar number of reports have found no correlation at all or a modest positive correlation, even for the same organism. Table 1 compiles transcriptome-wide analyses of mRNA decay in different organisms, techniques used, and information on the reported relationships between mRNA abundance and mRNA half-life.

The question has arisen if lower mRNA concentrations can actually cause their degradation to be slowed. This idea is suggested by an observation made by several groups, in several species, that in log phase growth, mRNA half-lives are inversely correlated with steady-state abundance (Figure 7). For example, a weak negative correlation was shown between mRNA concentration and mRNA half-life for *E. coli* cells in exponential phase (Bernstein et al., 2002). Stronger negative correlations were reported in *L. lactis* (Redon et al., 2005b), and in *M. tuberculosis* (Rustad et al., 2013), both in exponentially growing bacteria. Moreover, in the latter study the overexpression of genes in the DosR regulon resulted in transcripts with shorter half-lives. Other reports in *E. coli* and *L. lactis* showed that cells growing at different growth rates also show a negative correlation between these parameters (Dressaire et al., 2013; Esquerre et al., 2015). For example, changes in growth rate from 0.1 to 0.63 h^–1^ – using chemostats – resulted in increased mRNA levels and a decreased median mRNA half-life from 4.2 to 2.8 min, respectively (Esquerre et al., 2014, 2015). Transcription modulation using five constructs with distinct 5′ UTRs in *lacLM* mRNA also depicted a similar trend in *L. lactis* in exponential phase, and a similar outcome was obtained for *lacZ* in *E. coli*, using P_BAD_-mediated transcription regulation (Nouaille et al., 2017). Two of the studies described here (Rustad et al., 2013; Nouaille et al., 2017) reported inverse relationships between mRNA abundance and half-life in defined systems where expression was modulated by inducible promotors and growth rate was not affected. This strongly suggested that transcription rate can directly influence degradation rate. However, contradictory findings have been reported.

An *E. coli* transcriptome-wide mRNA half-life study by a different group reported that the rate of mRNA degradation had a very weak positive correlation with mRNA abundance for both exponential phase (*R*^2^ = 0.07) and stationary phase (*R*^2^ = 0.19) (Chen et al., 2015), in contrast to other *E. coli* studies (Bernstein et al., 2002; Esquerre et al., 2014, 2015). In *Bacillus cereus*, mRNA half-life had a positive correlation with expression level (Kristoffersen et al., 2012), while in *Stenotrophomonas maltophilia* and *Chlamydia trachomatis* trachoma and lymphogranuloma venereum biovars no correlations were found (Bernardini and Martinez, 2017; Ferreira et al., 2017). In *M. smegmatis*, induced overexpression of *dCas9* (in the absence of a gene-targeting sgRNA) did not alter its half-life in log phase (Vargas-Blanco et al., 2019). Surprisingly, overexpressing *dCas9* under hypoxic stress increased its mRNA stability by approximately twofold (Vargas-Blanco et al., 2019). Moreover, re-exposure of hypoxic *M. smegmatis* cultures to oxygen caused half-lives of several tested genes to immediately return to log-phase like levels, despite transcription being blocked by rifampicin and transcript levels therefore remaining low (Vargas-Blanco et al., 2019). Other reports have indicated that the relationship between mRNA abundance and half-life differs in various stress conditions. In carbon-starved *L. lactis* there was a positive correlation between mRNA degradation and abundance (Redon et al., 2005b), while the opposite was observed during isoleucine starvation (Dressaire et al., 2013). Work in eukaryotes suggests complexities that could conceivably occur in bacteria as well. In *S. cerevisiae*, under DNA damaging conditions, upregulated genes are usually stabilized and repressed genes are prone to degradation (Shalem et al., 2008). Conversely, under oxidative stress upregulated genes are destabilized, with the opposite scenario for repressed genes (Shalem et al., 2008). Furthermore, an in-depth analysis in that work revealed a trend between these two stress conditions: Genes with a rapid transcriptional regulation show a negative correlation between mRNA abundance and mRNA degradation. On the other hand, genes subject to a slow transcriptional response follow a positive correlation between mRNA abundance and degradation (Shalem et al., 2008).

Clearly, further work is needed to reconcile contradictory findings in bacteria with respect to the relationships between mRNA abundance and stability. Some reported differences may be attributable to differences between species, while others may result from differences in methodology for measuring half-life. Most studies measure half-life by measuring decreases in mRNA abundance following transcription blockage by rifampicin. Variability may arise from the time-points chosen to assay abundance following transcriptional block, given that we and others have reported multiphasic decay kinetics (Hambraeus et al., 2003; Selinger et al., 2003; Chen et al., 2015; Nguyen et al., 2020). Methodology for normalization and for calculating half-lives also vary (see Table 1).

## The Importance of RNA Decay in Clinically Important Species

Pathogenic bacteria have developed mechanisms that allow them to survive often-hostile host environments by sensing cues and mounting specific responses at both transcriptional and posttranscriptional levels. These pathogens exhibit highly specific responses to some stressors, as well as broader responses to conditions such as energy stress, where resources are preserved by global modulation of processes including translation, protein degradation, transcription, and RNA stabilization (Bohne et al., 1994; Sherman et al., 2001; Park et al., 2003; Christiansen et al., 2004; Wood et al., 2005; Papenfort et al., 2006; Liu et al., 2010; Fritsch et al., 2011; Galagan et al., 2013; Guo et al., 2014; Sievers et al., 2015; Quereda et al., 2018; Ignatov et al., 2020).

In *L. monocytogenes*, PrfA serves as a transcriptional regulator of multiple virulence factors, such as phospholipases PlcA and PlcB, and the toxin listeriolysin O (Leimeister-Wachter et al., 1990, 1991; Quereda et al., 2018). Expression of PrfA itself is regulated by several mechanisms at the translational and transcriptional level. For example, PrfA translation is temperature-regulated by a stem-loop in its transcript, *prfA*, that prevents ribosome access to the SD sequence at 30°C but not at 37°C (Johansson et al., 2002). *prfA* is also regulated by an S-adenosylmethionine riboswitch and its product, the sRNA SreA, that blocks translation after binding the 5′ UTR (Loh et al., 2009). Additionally, while the stem-loop increases *prfA* stability (Loh et al., 2012), the binding of SreA to *prfA* triggers transcript degradation (Loh et al., 2009). Also in *L. monocytogenes*, posttranscriptional regulation of Tcsa, the T cell-stimulating antigen encoded by *tcsA*, was recently reported to be under the control of the sRNA LhrC in a translation-independent manner, by recruiting an undefined RNase (Ross et al., 2019). In *S. aureus* SarA, a histone-like protein, influences mRNA turnover of virulence factors, such as protein A (*spa*) and the collagen adhesion protein (*cna*) during exponential growth (Roberts et al., 2006; Morrison et al., 2012). Also in *S. aureus*, the multifunctional RNAIII binds other RNAs, recruiting RNase III to initiate transcript degradation. Some of RNAIII’s targets are *spa*, *coa* (encoding coagulase), *sbi* (encoding the IgG-binding protein Sbi), and *SA1000* (encoding the fibrinogen-binding protein SA1000) (Huntzinger et al., 2005; Boisset et al., 2007; Chevalier et al., 2010), playing an important role in *S. aureus* virulence and response to stress. In *S. enterica*, under low Mg^2+^ conditions synthesis of the antisense AmgR RNA leads to interaction and destabilization of the *mgtC* transcript (encoding the virulence protein MgtC), in an RNase E-dependent manner (Lee and Groisman, 2010). Hence, regulation of the stabilities of specific mRNAs has a major role in the survival and virulence responses of pathogens.

Recent reports have suggested unexpected relationships between RNases and drug resistance. Nonsense and INDEL mutations in *Rv2752c*, encoding RNase J, were associated with drug resistance in a GWAS study that identified resistance-associated mutations in whole-genome sequences of hundreds of *M. tuberculosis* clinical isolates (Hicks et al., 2018), as well as an earlier study performing similar analyses on a smaller set of clinical isolates (Zhang et al., 2013). Another study, reporting whole-genome sequences of 154 *M. leprae* clinical isolates from 25 countries, found a disproportionately high number of polymorphisms in *ML1040c*, encoding RNase D, and *ML1512c*, encoding RNase J (Benjak et al., 2018). These mutations were not directly associated with drug resistance, but appeared to be under positive selection (Benjak et al., 2018).

Global mRNA stabilization is another feature associated with bacterial stress response and non-growing conditions (see Table 1). Cells in quiescent states contain relatively low levels of mRNA, with greatly reduced transcriptional and translational activity (Betts et al., 2002; Wood et al., 2005; Kumar et al., 2012; Rittershaus et al., 2013). In some cases, these states share similarities with *B. subtilis* spores, in which the bacteria have dramatically reduced mRNA turnover (Segev et al., 2012). This can be interpreted as a concerted cellular effort to downregulate global gene expression and preserve cellular resources, until encountering a suitable environment to resume growth. At the same time, having paused translational machinery may permit allocation of resources toward specific responses needed to survive a given condition, such as those described in the previous paragraph. Importantly, stress responses that establish and maintain non-growing states not only allow pathogens to survive these stressors, but also induce broad antibiotic tolerance, since most antibiotics are relatively ineffective at killing non-growing cells (for example, Rao et al., 2008). This relationship between growth arrest and antibiotic tolerance may be one of the reasons why months of multidrug therapy are required to prevent relapse in tuberculosis patients, where large numbers of bacteria are likely semi-dormant in hypoxic granulomas (Garton et al., 2008). The apparent universality of mRNA stabilization as a response to energy stress and other stressors that inhibit growth, compared to gene-specific mRNA regulation, brings up fascinating possibilities as a prospective target for therapeutic development. There has been a surge in antimicrobial resistance in recent decades, prompting collaborative efforts between academia and industry to develop new antimicrobials (Ventola, 2015a,b; World Health Organization [WHO], 2019). As we approach an understanding of the mechanisms behind mRNA turnover – and strive to unveil how transcript fate is regulated under stress conditions – we would like to emphasize the essentiality of mRNA degradation in bacteria, and the roles of RNases in the virulence and survival responses of pathogens. Many clinically important antibiotics target transcription and translation, highlighting the potential of targeting these central dogma processes from the opposite angle. In early steps in this direction, a protein degradation inhibitor was found to have strong activity against mycobacteria (Gavrish et al., 2014) and inhibitors of RNase E have been reported (Kime et al., 2015).

## Conclusion

Transcriptome stabilization as a stress response is widespread across the bacterial domain. This globally concerted response is implicated in gene regulation and survival, as well as pathogenesis in bacteria. We have described and discussed various mechanisms of mRNA degradation and stabilization, many of which have established roles in regulation of specific genes, but have not yet been able to explain transcriptome-wide half-life alterations. We hope that the information presented here helps to inspire further study that will uncover the mechanism(s) behind global transcriptome stabilization in stress, which so far remains elusive. Finally, we hope to inspire the reader to find these mysteries as scientifically stimulating as we do.

## Author Contributions

DV-B and SS wrote the manuscript. Both authors contributed to the article and approved the submitted version.

## Conflict of Interest

The authors declare that the research was conducted in the absence of any commercial or financial relationships that could be construed as a potential conflict of interest.
